# Bright and fast scintillations of an inorganic halide perovskite CsPbBr_3_ crystal at cryogenic temperatures

**DOI:** 10.1038/s41598-020-65672-z

**Published:** 2020-05-25

**Authors:** V. B. Mykhaylyk, H. Kraus, V. Kapustianyk, H. J. Kim, P. Mercere, M. Rudko, P. Da Silva, O. Antonyak, M. Dendebera

**Affiliations:** 1 Diamond Light Source, Harwell Campus, Didcot, OX11 0DE UK; 2 University of Oxford, Department of Physics, Denys Wilkinson Building, Keble Road, Oxford, OX1 3RH UK; 3 Scientific-technical and Educational Centre of low Temperature Studies, I. Franko National University of Lviv, 50 Dragomanova str., 79005 Lviv, Ukraine; 4 Department of Physics of Kyungpook National University, 1370 Sangyeok-dong, Buk-gu, Daegu, 702-701 South Korea; 5 Synchrotron Soleil, L’Orme des Merisiers, Saint-Aubin, BP 48, 91192 Gif-sur-Yvette, France; 6 I. Franko National University of Lviv, Physics Department, 8 Kyrylo and Mefodiy str., 79005 Lviv, Ukraine

**Keywords:** Materials science, Materials for devices

## Abstract

Highly efficient scintillation crystals with short decay times are indispensable for improving the performance of numerous detection and imaging instruments that use- X-rays, gamma-quanta, ionising particles or neutrons. Halide perovskites emerged recently as very promising materials for detection of ionising radiation that motivated further exploration of the materials. In this work, we report on excellent scintillation properties of CsPbBr_3_ crystals when cooled to cryogenic temperatures. The temperature dependence of luminescence spectra, decay kinetics and light yield under excitation with X-rays and α-particles was investigated. It is shown that the observed changes of spectral and kinetic characteristics of the crystal with temperature can be consistently explained by radiative decay of free excitons, bound and trapped excitons as well as electron-hole pairs originating from their disintegration. It has been found that the crystal exhibits a fast decay time constant of 1 ns at 7 K. The scintillation light yield of CsPbBr_3_ at 7 K is assessed to be 50,000 ± 10,000 ph/MeV at excitation with 12 keV X-rays and 109,000 ± 22,000 ph/MeV at excitation with α-particles of ^241^Am. This finding places CsPbBr_3_ in an excellent position for the development of a new generation of cryogenic, efficient scintillation detectors with nanosecond response time, marking a step-change in opportunities for scintillator-based applications.

## Introduction

Halide perovskites have been around for a long time and currently they receive significant attention from a range of research areas. The first investigation of photoconductivity in CsPbX_3_ (X = Cl, Br or I) was carried out in 1958^1^. Following this, studies of structure, optical, and luminescence properties were published over the course of half a century, demonstrating steady but rather low interest in these materials^2–5^. A step change of interest in the halide perovskite family has occurred during the last decade as a result of rapid evolution of solid-state photovoltaic devices based on hybrid organic metal halide perovskites - materials with the general formula MAPbX_3_, where MA = methyloamine CH_3_NH_3_, and X = Cl, Br, or I^6,7^. It was realised quickly that the remarkable physical properties of perovskites made them highly attractive for optoelectronic applications. Specifically, the outstanding high quantum yield of photoluminescence of these materials is a key feature enabling bright light emitting devices and lasers^8^, whereas the high current conversion efficiency underpins their application as photodetectors and solar cells^9^.

Because of the high atomic number of Pb, Br and I atoms, hybrid organic metal halide perovskites exhibit enhanced X-ray stopping power. That, in combination with the large mobility of charge carriers, makes them very attractive for application in radiation detection. These devices convert the energy deposited by X-ray photons into a change of electric current. The first detection of soft X-rays (<10 keV) using the photoelectric effect in polycrystalline MAPbI_3_ films was demonstrated in^10^. The necessity of improving the detection efficiency of hard X-rays (>100 keV) saw the initiation of the development of X-ray detectors based on single crystals of hybrid organic metal halide perovskites^11–13^. The energy spectra measured with these detectors demonstrated a respectable energy resolution of 35% for 59.6 keV ^241^Am^11^ and 6.5% for 662 keV of ^137^Cs γ-radiation^13^. The success of hybrid organic metal halide perovskites triggered an increase of research activity on their all-inorganic counterparts, which offer even higher X-ray absorption and much better chemical stability. These efforts resulted recently in the advent of a single crystal CsPbBr_3_ detector for hard X-rays and gamma quanta with excellent energy resolution of 3.8% at 662 keV, which is comparable with the capability of commercially available detectors^14^. The results show that inorganic halide perovskites are very promising materials for detecting ionising radiation. These achievements provided fresh motivation for further research into halide perovskites for radiation detection, in particular exploring their potential as scintillation detectors.

The idea of harnessing the fast radiative decay of excitons in halide perovskites for scintillation detection has been around for long, basically since the first observation of very fast and intense X-ray luminescence of excitons in CsPbX_3_ (X = Cl, Br, I) at low temperature in 1993 by Voloshynovskyi *et al*.^15^. However, the light yield of single crystal scintillators at room temperature was found to be very low, i.e. <500 ph/MeV^16^ rendering these materials uninteresting for scintillation detection at room temperature. Research into this area received a fresh impetus when sub-nanosecond scintillation decay with a relative light output of 11% compared to that of NaI-Tl (5500 ph/MeV) at room temperature was found in the layered organic metal-halide (C_6_H_13_NH_3_)_2_PbI_4_ ^17^. As mentioned above, advances in radiation detector technology based on hybrid organic metal halide perovskites provided new inspiration for these studies. Subsequently light yields of 9000 ph/MeV and 14000 ph/MeV respectively were reported in other layered organic metal halide perovskites, i.e. (EDBE)PbCl_4_ (EDBE = 2,2’-ethylenedioxy)-bis(ethylamine))^18^ and (C_6_H_5_C_2_H_4_NH_3_)_2_PbBr_3_ ^19^, highlighting the potential of these materials for scintillation detectors. The combination of extensive research in this field and advances in the fabrication of CsPbX_3_ nanocrystals resulted recently in an important technological breakthrough – the development of a highly sensitive scintillation screen for X-ray imaging^20–23^.

These achievements also prompted renewed interest in studies of scintillation properties of inorganic perovskite single crystals. It is worth pointing out that scintillation light yield is inversely proportional to the band gap energy so that in the absence of thermal quenching, narrowband halide perovskites offer a theoretical maximal light yield that could exceed by more than a factor of two what is observed in the best modern scintillators^24,25^. Applications of scintillation detectors at cryogenic temperatures in scientific research are on the increase; moreover, the explorations have started to assess the feasibility of fast and bright cryogenic scintillators for nuclear imaging^26^. It is this growth in importance that motivated us to characterize the scintillation of CsPbBr_3_ crystals over the 7–200 K temperature range. In this work we carried out temperature-dependent measurements of X-ray luminescence, decay time and scintillation light yield of CsPbBr_3_. Exploring properties as function of temperature, we aim to identify possible applications of this material as a cryogenic scintillation detector. Furthermore, such studies allow deeper insight into the physical processes that occur in perovskite materials in general, thus enabling further optimization.

## Results and Discussion

**Figure 1:**
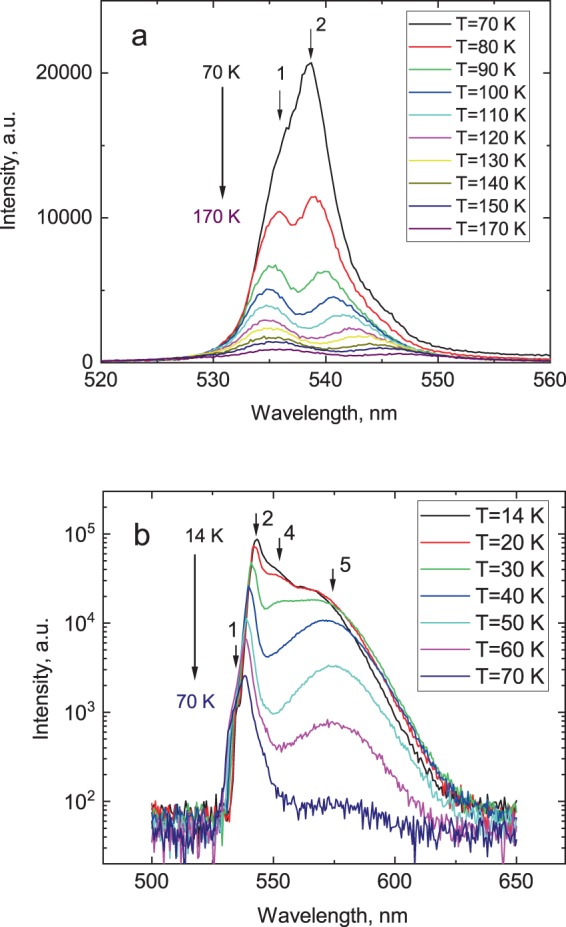
X-ray luminescence spectra of a CsPbBr_3_ crystal measured at different temperatures. For better visualisation of changes the spectra are presented for two ranges of temperatures (**a**) 70–170 K (in linear scale) and (**b**) 14–70 K (in logarithmic scale).

The use of materials as scintillators relies on their ability to convert the high-energy excitation into visible emission. Luminescence is the final stage in the chain of energy transformation processes and studies of the luminescence properties are essential. Therefore, we begin by presenting the results on luminescence of CsPbBr_3_ as a function of temperature. Figure 1(a,b) display the temperature-dependant X-ray luminescence spectra measured under steady state X-ray excitation for temperatures between 12 and 170 K. Results of these measurements show that the X-ray luminescence spectrum of the single crystal manifests very pronounced temperature changes both in intensity and overall shape.

**Figure 2:**
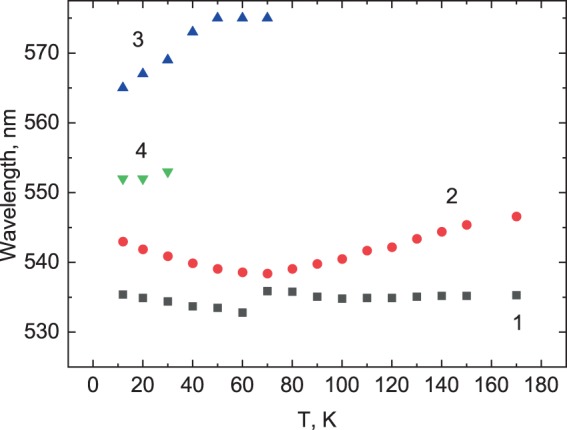
Evolution of the emission peaks in CsPbBr_3_ with temperature.

Two emission peaks at 535 nm (peak 1) and 545 nm (peak 2) appear when the temperature decreases below 200 K as depictured in Fig. 1a. Crystal cooling leads to a gradual increase of the peak intensities. Initially, the position of the short wavelength peak 1 shows very small changes while peak 2 shifts towards higher energies, so that below 70 K they amalgamate and only one band dominates in this part of the spectrum (see Fig. 1a). When the temperature of the crystal reduces below 70 K the steady pattern of evolution of the luminescence spectra experiences sweeping transformation. The intensity of peak 2 begins to rise very rapidly with cooling while direction of the peak shift reverses towards lower energies. Interestingly, a shoulder-like feature in the short-wavelength part of the spectrum related to unresolved peak 1, demonstrates the same red shift with decrease of temperature. This can be seen in the Fig. 2 where the overview of temperature dependent shift of the individual emission peaks is presented. It is plausible that the mentioned above changes observed around 70 K are indication of temperature-induced changes in the mechanism, which is responsible for the emission in this spectral range. Additional distinctive changes in the luminescence of CsPbBr_3_ are new bands appearing in the long-wavelength part of the spectrum. Initially this is a broad band at 575 nm (peak 3) and then, when temperature reduces to 30 K, a new peak 4 at ca. 555 nm emergees in the X-ray luminescence spectra of the crystal.

The luminescence properties of CsPbBr_3_ have been the subject of numerous studies over two decades. It has been suggested then that short-wavelength emission peaks originate from the radiative decay of free and bound (localised) excitons^2,4,5,15^. The excitonic nature of CsPbBr_3_ emission, which has been supported later by extensive studies^27–33^ is currently widely accepted. Very recently, this emission attracted a great deal of attention that lead to extended interpretation. The peaks were assigned to the emission of excitons and bi-excitons^34^ or direct and indirect transitions from two minima in the conduction band states^32,35^. This feature of the band structure, called Rashba-splitting, is due to strong spin-orbit coupling and breaking the inversion symmetry in the direction perpendicular to the k-vector of the Brillouin zone of the crystal.

The luminescence of CsPbBr_3_ manifests marked changes with temperature, type and power density of excitation that may explain the discrepancies in interpretation and assignments of individual emission bands. Furthermore, the luminescence of CsPbBr_3_ depends on the size of the crystals and their quality which makes the identification and interpretation of emission properties even more complicated. When the dimensions of a crystal decrease to tens of nm, the quantum confinement phenomenon leads to drastic changes: a fine structure appears at the high-energy side of the emission spectra^36–38^, the rate of the emission kinetics accelerates^31–33,39^ and, importantly, it exhibits no thermal quenching with the quantum yield exceeding 90% at room temperatures^30,40,41^. It is worthwhile noting that these remarkable features underpin the excellent performance of optoelectronic devices employing CsPbBr_3_ nanocrystals^21,42–44^. The quantum confinement effects are proven to cause a dramatic alteration of the luminescence properties of the material under study. Therefore, throughout this work, where the emphasis is on properties of bulk CsPbBr_3_ single crystals, we will make pertinent references to the nanomaterials when relevant.

The luminescence intensity of bulk crystals grown from melt or solution varies by orders of magnitude^45^ and is thermally quenched at T > 200 K^46,47^. Earlier measurements of bulk CsPbBr_3_ single crystals reported three emission bands at 533 nm 548 and 573 nm^4,5^ at T < 10 K. The high energy luminescence peak 1 at ca. 533 nm observed in the studies of CsPbBr_3_ single crystals at low temperatures is unanimously attributed to the emission of free excitons^4,5,15,27,29,36,46–48^. The contribution of this peak to X-ray luminescence is negligible below 70 K, for the high-energy part of the emission spectrum is dominated by strong peak 2. Nonetheless, at higher temperatures the intensities of both peaks are comparable. Peak 2 can be attributed to the emission of excitons bound to a halide vacancy as was suggested in^28^. The peak exhibits a pronounced power law dependence of emission intensity on the excitation intensity of photoexcitation that is characteristic for excitonic processes^49,50^. Therefore, depending on excitation density this peak may amalgamate with the short-wavelength peak^34,51^. This effect, combined with a thermal shift, might be an explanation for this emission band, reported with a range of values, i.e. 540^28^, 543^47^ 548 nm^51^.

The temperature dependence of the edge luminescence in semiconductors follows the changes of the bandgap that in turn reflects a combined effect of the change in the crystal lattice and electron-phonon interaction. While the lattice contraction with cooling leads to the blue shift, the electron-phonon-interaction has the inverse effect that can cause anomalous red shift. In halide perovskites, the second effect is believed to control the band gap behaviour^51,52^ that can explain the coherent red shift of exciton peaks 1 and 2 observed in CsPbBr_3_ at low temperatures. However, at higher temperatures (T > 70 K), peak 2 exhibits the opposite trend. A similar pattern in the luminescence of CsPbBr_3_ crystals at higher temperature has been reported recently^35^. This observation may indicate the changes in the cause of the underlying emission mechanism at higher temperature due to, for instance, delocalisation or disintegration of the excitons. In this case, the observed feature may be explained as transition from exciton recombination to radiative recombination of free electrons and holes. Such changes in the emission mechanism will influence the temperature shift of emission bands in the luminescence spectra^53^.

**Figure 3:**
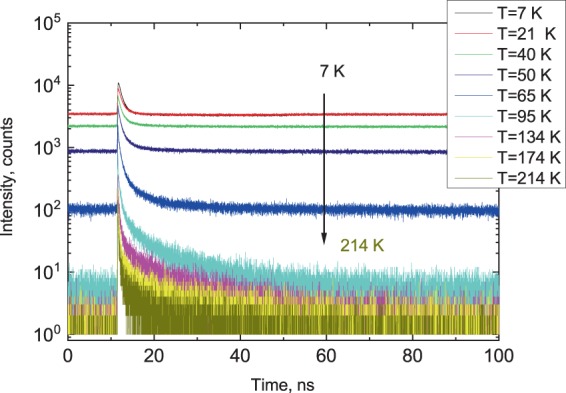
Decay curves of X-ray luminescence measured in the CsPbBr_3_ crystal as temperature is changed from 214 down to 7 K. The luminescence is excited by 12 keV X-ray pulses of synchrotron radiation.

The long-wavelength luminescence band at 575 nm (peak 3), observed at low temperature in the emission spectra of CsPbBr_3_ single crystals produced from the melt^5,29,48^ is much weaker in the solution-grown crystals^47^. Importantly, no emission in this region has been reported for CsPbBr_3_ nanocrystals. Taking into account that the concentration of defects is less in solution-grown crystals and it is further reduced in nanocrystals we attribute this band to the emission of trapped excitons or carriers. It has been postulated that this emission is caused by energy transfer from excitons^5^ but the exact nature of the trapping centres remains to be determined. The peak 4 emerging at 555 nm in the CsPbBr_3_ crystal cooled below 30 K may be attributed to the emission of self-trapped excitons^54^. Next, we studied the scintillation kinetics of CsPbBr_3_ over the low-temperature range using pulsed X-ray excitation. It should be noted that decay curves measured for excitation with X-rays are usually different from those observed for optical excitation. This difference is readily explained by the fact that X-rays produce ionisation tracks with a high density of charged particles, which then transfer excitation energy to donor-acceptor pairs and excitons. Figure 3 shows the complex character of changes observed in the scintillation decay curves of the CsPbBr_3_ crystal with cooling. The decay curves exhibit clear non-exponential kinetics, which is indicative of the superposition of a few emission processes^55–57^. The prominent features of CsPbBr_3_ decay curves are (i) the rapid change of peak intensity of the luminescence signal with cooling and (ii) the emergence of background below 70 K, suggesting the existence of a very slow recombination process. These features can be related to the concomitant changes in the X-ray luminescence spectra of the crystal (see Fig. 1a,b). The first one correlates with the overall increase of emission intensity observed for the 535 and 545 nm peaks. These two bands give rise to the fast nanosecond decay in the luminescence pulse. The second feature is consistent with the increase of the long wavelength emission band, having the decay time constant on the microsecond timescale^5^. It is therefore sensible to attribute the background observed in the scintillation decay curves of CsPbBr_3_ below 70 K to the very slow radiative decay of carriers released from traps.

**Figure 4:**
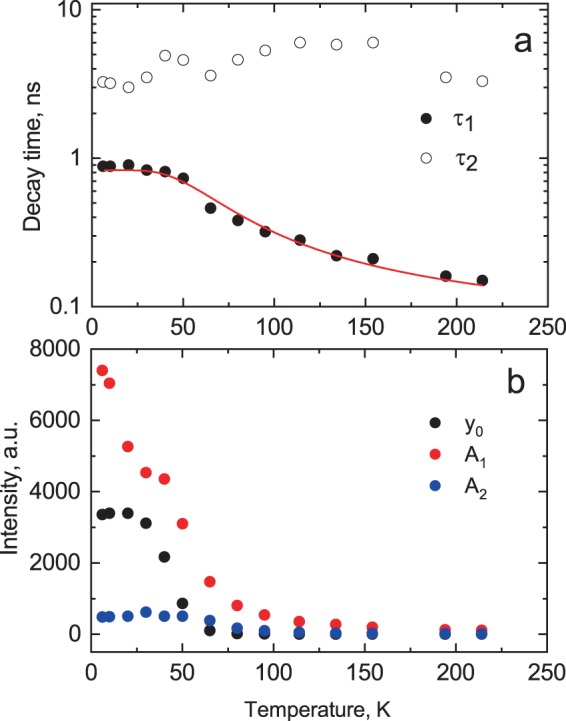
Temperature dependence of decay constants (**a**) and amplitudes plus background (**b**) obtained from the fitting of the decay curves of CsPbBr_3_ with a sum of two exponential functions: $y={A}_{1}\exp (-t/{\tau }_{1})+{A}_{2}\exp (-t/{\tau }_{2})+{y}_{0}$. The line in (**a**) shows the best fitting of the temperature dependence of the fast decay constant τ_1_ = ƒ(T) using the model of thermally activated transition over energy barrier $1/{\tau }_{1}=1/{\tau }_{0}+Kexp(-\varDelta E/kT)$. The parameters of fit for τ_1_ = ƒ(T) are: ${\tau }_{0}$ = 1.0 ± 0.01 ns, *K* = 12.8 ± 1.6 × 10^9^ s^−1^, Δ*E* = 15 ± 1 meV.

The measured decay curves were fitted using the sum of two exponentials and a constant: $f(t)={A}_{1}\exp (-t/{\tau }_{1})+{A}_{2}\exp (-t/{\tau }_{2})+{y}_{0}$ where ${y}_{0}$ is background, ${A}_{1,2}$ and ${\tau }_{1,2}$ are the amplitudes and decay time constants of the two emission components, respectively. Note that the fit to a sum of two exponentials is widely applied to luminescence decay curves in semiconductors in general^58–60^ as well as organic^61,62^ and inorganic perovskites^33,38^. We concluded that two exponentials plus a constant background provided an adequate representation of the measured decay curves over a broad temperature range. The decay time constants and amplitudes obtained from the fitting procedure are displayed in the Fig. 4 as functions of temperature.

Further details and trends in the temperature evolution of the luminescence kinetics of CsPbBr_3_ crystals, which are rather different from those reported for nanocrystals, can be deduced from the analysis. In literature, the two temporal components in the emission of nanostructures in semiconductors are attributed to the decay of bright and dark excitons^59,63–65^. This model has recently been used for the interpretation of temperature dependence of luminescence dynamics of CsPbX_3_ nanocrystals^33^. Only the emission band of free excitons dominates in the luminescence spectra of CsPbBr_3_ nanocrystals due to quantum confinement^30^ that permits the separation of contributions from the bright and dark states of the free excitons in the decay curves^33^. On the contrary, the integrated decay kinetics of the bulk crystal measured by us over the whole luminescence spectrum range represents a superposition of several emission processes that cannot be isolated.

Nonetheless, it is still possible to identify a few features in the luminescence pulse that can be assigned to a specific decay channel. As can be derived from Fig. 4a, the first decay time constant exhibits a gradual increase with cooling but below 30 K it settles at ${\tau }_{1}$ = 1 ns. The amplitude of this component also increases very steeply with cooling, as displayed in Fig. 4b. This temperature dependence of the decay rate is very typical for emission that is governed by thermally activated depopulation of excited states^66–69^. We therefore attribute this decay component to the emission of bound excitons. The temperature dependence of the fast decay time constant (${\tau }_{1}$) can be modelled by using the well-known expression for thermally-activated non-radiative processes as $1/{\tau }_{1}=1/{\tau }_{0}+Kexp(-\varDelta E/kT)$, where *τ*_0_ is the luminescence decay time constant at T = 0 *K* is the rate of deactivation, Δ*E* is the thermal activation energy, and *k* is Boltzmann’s constant. The best fit of the data displayed in Fig. 4a yields value of Δ*E* = 15 ± 1 meV, which is in good agreement with the energy of LO phonons in CsPbBr_3_ (19 meV) reported in^4^ and^70^. Within the framework of the model this value represents an amount of energy that is needed for exciton delocalization.

At higher temperature when the bound excitons can escape the centre of localisation due to thermal activation, the emission due to free excitons becomes more pronounced^55,68^. This also correlates with the behaviour of a second decay component (${\tau }_{2}$) and the peak in the short-wavelength part of the luminescence spectra. Consequently, this decay component may be assigned to the emission of free excitons. Very slow emission, manifesting itself below 70 K as a steady background in the decay curves, is due to radiative recombination of self-trapped excitons or excitons captured by defects or impurities. The excitons trapping is much enhanced in bulk crystals where free excitons can travel a long distance before they find a trapping centre as opposed to nanostructures where exciton diffusion is limited by the quantum confinement effect. Nonetheless, the contribution of background is negligible above 100 K, where fast sub-nanosecond emission dominates in the radiative decay of CsPbBr_3_ crystal.

**Figure 5:**
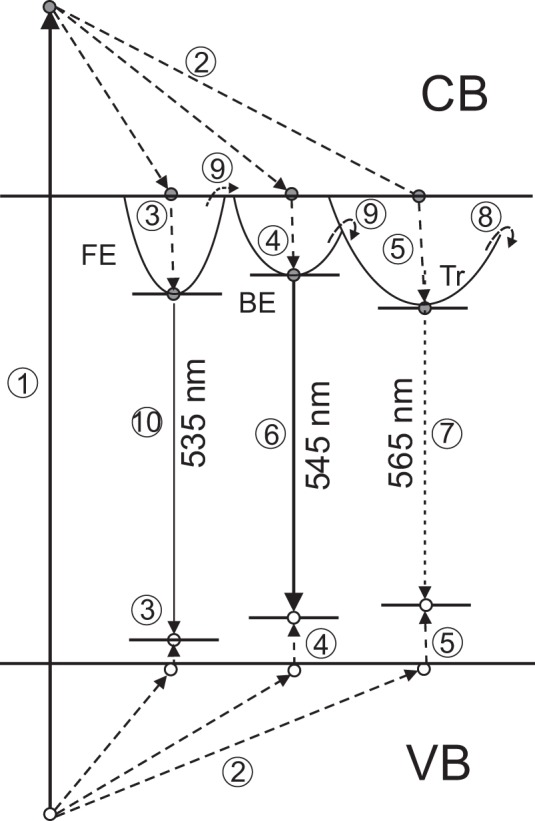
Proposed scheme of emission processes in CsPbBr_3_ crystals that explains the observed temperature dependences. 1- X-ray photon creates free carriers in the valence (VB) and conduction band (CB), 2 – relaxation of electrons and holes, 3, 4– formation of free (FE) and bound (BE) excitons, 5- carrier captured by traps (Tr), 6- fast emission of bound excitons, 7 -very slow recombination of trapped charge carriers, 8 –thermally activated non-radiative quenching, 9- temperature dependent delocalization and fusion of free and bound excitons, 10- emission of free excitons.

Following these findings and arguments, we propose a consistent model that explains the temperature dependences of the spectral and kinetics properties of luminescence observed in CsPbBr_3_ crystals. The hot carriers created by ionising radiation (process 1) promptly relax to the bottom of the conduction band (electrons) and the top of the valence band (holes) (2) as displayed schematically in Fig. 5. At very low temperatures the electrons and holes create free excitons (3) excitons bound to the halide vacancies (4) or they are captured by deeper traps (5). Both free and bound excitons recombine promptly with characteristic decay time ca.1 ns, giving rise to the fast component in the decay curve and a short wavelength emission with dominant peak at 545-nm in the luminescence spectra (6). The probability to recombine radiatively is very low for the trapped excitons, and that leads to a very slow emission bands around 565 nm (7).

As the temperature increases, the trapped excitations begin to decay non-radiatively (8) which causes the decrease of background contribution to the scintillation pulse and intensity of the long-wavelength emission bands. The bound excitons also begin to delocalise (9) and disintegrate. The delocalised excitons or related electron-hole pairs can subsequently decay through the non-radiative channel (8). This correlates with the gradual decrease of the intensity of the pertinent peak in the emission spectra and amplitude of the fast component in the decay curves.

Above 70 K, when all the traps are emptied, the very slow recombination luminescence (7) vanishes; no indication of this emission is seen in the luminescence spectra or decay curves. Instead, the decay curves and luminescence spectra of CsPbBr_3_ crystals manifest only the characteristic features of the emission of free (10) and bound excitons (6). The decrease of the emission efficiency at these temperatures is primarily caused by the excitons disintegration (9) that is followed by non-radiative recombination processes (8).

These experiments demonstrated that CsPbBr_3_ exhibits a fast scintillation response under excitation with X-rays at low temperatures. Assessment of the scintillator performance requires knowledge of the light yield of the material and to do such evaluation, we used two approaches. In both cases, the scintillation light yield of the crystal under study was derived through the comparison with the reference LYSO-Ce scintillator as described in the Method section.

**Figure 6:**
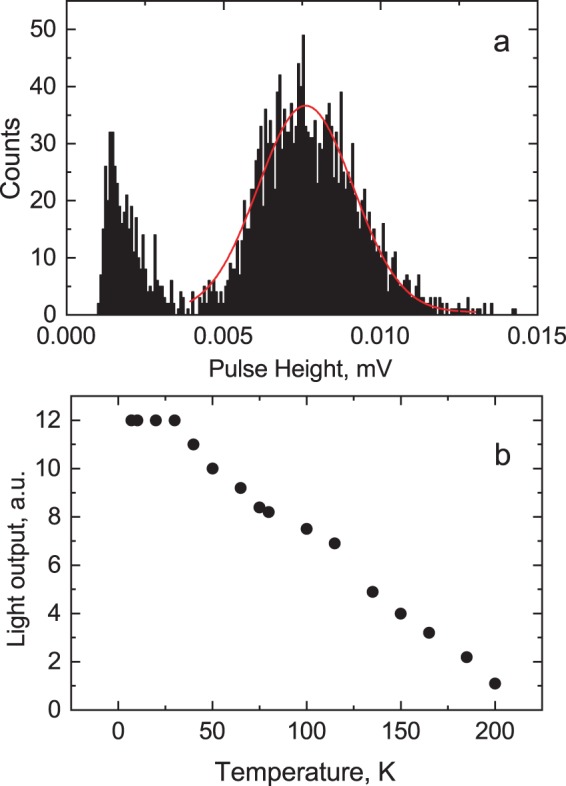
(**a**) Pulse height spectrum of scintillations excited through α-particle interaction from ^241^Am in CsPbBr_3_ at 7 K. The pulse height distribution that signifies scintillation response due to α-particles is fitted by a Gaussian (red line). (**b**) Light output of CsPbBr_3_ as function of temperature for α-particle excitation (^241^Am).

Figure 6a shows the pulse height spectrum measured at 7 K that features a peak with Gaussian shape, attributed to 5.5 MeV α-particles emitted by an ^241^Am source and detected by the CsPbBr_3_ crystal. The position of the peak centre is used as a measure of scintillation light output at different temperatures, as displayed in Fig. 6b. The temperature dependence was fitted using Mott’s equation $I={I}_{0}/(1+Kexp(-\varDelta E/kT)$, where *I*_0_ is the initial luminescence intensity, *K* is a constant, $\varDelta E$ is the thermal activation energy of non-radiative decay and *k* is Boltzmann’s constant. The characteristic energy that activates the non-radiative recombination processes in CsPbBr_3_ is found to be 10 ± 1 meV that is slightly different from the value obtained from the fitting the temperature dependence of the fast decay constant. The discrepancy can be readily understood, as here this parameter is the effective activation energy determined from integrated scintillation response that originates from the contribution of different emission components.

A clearly measurable scintillation response is detected when the crystal is cooled to below 200 K. Thus, by taking the light yield of LYSO-Ce equal to 34000 ph/MeV, the light yield of CsPbBr_3_ was determined as 109,000 ± 22,000 ph/MeV at 7 K. It is also pertinent to remark that the theoretical estimate made under the assumption of no thermal quenching following the semi-empirical approach^71,72^ predicts a scintillation light yield of CsPbBr_3_ as 117,000 ph/MeV (see Experimental technique section). Such convergence of experimental findings with theoretical estimates is reassuring and encouraging.

**Figure 7:**
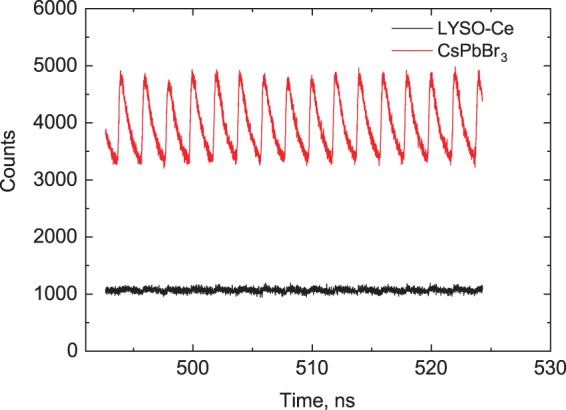
The sequence of X-ray pulses from the synchrotron detected using CsPbBr_3_ (T = 7 K, red) and LYSO-Ce (T = 292 K, black) scintillators. The graphs demonstrate the superior timing resolution of the scintillation response of CsPbBr_3_ crystal as opposed to the nearly featureless signal from LYSO-Ce.

The sequences of scintillation pulses detected in CsPbBr_3_ and LYSO-Ce at X-ray excitation from synchrotron are displayed in Fig. 7. After integration over the time window and correction for the spectral response of the detector the light yield of CsPbBr_3_ under X-ray excitation was found to be 50,000 ± 10,000 ph/MeV at 7 K. Such noticeable reduction of the light yield at excitation with low energy X-rays is due to the non-proportionality effect which is a generic feature of scintillation detectors^73^.

**Figure 8:**
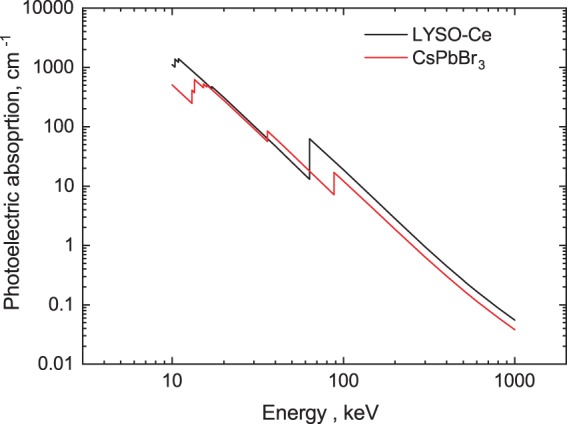
Photoelectric fraction of gamma-rays absorption in CsPbBr_3_ and LYSO-Ce. The data are calculated using the XCOM web-tool^88^.

A further requirement for a scintillator is that it exhibits a high absorption coefficient for high-energy ionizing radiation. This parameter scales roughly with the effective atomic number, and with CsPbBr_3_ being composed of heavy atoms, the absorption coefficient is high. The stopping power of CsPbBr_3_ crystal is competitive with the champions in the field as is evidenced in the Fig. 8 where the energy dependence of the photoelectric absorption coefficient in CsPbB_3_ and LYSO-Ce is shown.

## Conclusion

In this work we measured the scintillation decay time constant and light output of CsPbBr_3_ crystals down to temperature of 7 K and found that at low temperatures CsPbBr_3_ is a very bright scintillator with a rapid response, exhibiting a fast decay time constant of 1 ns at 7 K. The light yield is estimated as 50,000 ± 10,000 ph/MeV at excitation with 12 keV X-rays and 109,000 ± 22,000 ph/MeV at excitation with α-particles of ^241^Am. The latter value is close to what has been observed in MAPbBr_3_ single crystals^62^ approaching a theoretical limit for absolute light yield of CsPbBr_3_ crystal (117,000 ph/MeV). These findings highlight that CsPbBr_3_ is a very potent scintillation material for cryogenic applications. Examples of such applications are found in particle physics experiments where cryogenic scintillators are extensively used^74,75^. Further applications can be identified in space missions and nuclear medicine where a cryogenic environment is readily available. The additional efforts needed in order to provide a low-temperature environment for the operation of such detectors are likely to be compensated by potential benefits from using better scintillator. There are still challenges in optimising the crystal quality and developing the technology to produce crystals with reproducible characteristics. Currently the intense slow component observed below 70 K is an obvious disadvantage for scintillation applications as it causes afterglow. However, since we established the cause of this emission as such due to impurity or defect trapping centres, we also believe that there are possibilities to suppress or eliminate this emission through material adjustment using optimized growth processes^76,77^. Experts in scintillator production can achieve impressive level of improvements when pursuing a specific aim as has been demonstrated earlier^78^ and this approach can always be tried again for a new material.

## Methods

The CsPbBr_3_ crystal was grown from a stoichiometric mixture of CsBr and PbBr_2_ using the Bridgeman technique. The samples of volume 5 × 5 × 1.5 mm^3^ used in the measurements were cut from an ingot and polished. The sample was placed into a closed-cycle He cryostat, equipped with a DE-202A cryocooler (Advanced Research Systems) and Cryocon 32 (Cryogenic Control Systems Inc.) temperature regulator. The X-ray luminescence was excited by a URS-55A X-ray tube with a Сu-anticathode tube operating at 55 kV and 10 mA. The emission spectra were measured using a monochromator MDR-12 with photomultiplier module Hamamatsu H9305.

The scintillation decay curves of the crystals were investigated at Soleil synchrotron using a 12 keV monochromatic X-ray beam from the synchrotron. The measurements were carried out in hybrid mode, that is suitable for time-resolved experiments^79^ by triggering on a single X-ray pulse with FWHM of Δt = 47 ps, separated from the following pulse by a 147 ns gap. The sample attached to the holder was placed in a continuous-flow, He-cryostat (Oxford Instruments). The sample temperature was monitored using Si-diode sensor and stabilised by PID controller. The cryostat was attached to an XYZ-translation stage to facilitate alignment. The X-ray beam was impinging upon the sample placed at 45° to the incoming radiation through the 0.2 mm thick aluminised Mylar window. The luminescence from the illuminated area 1 × 1 mm^2^ was collected in reflection mode at 45° through a quartz window. The emission was detected in time-correlated single photon counting regime by a ID100 single photon counting detector.

The multiphoton counting technique^80^ was used to measure scintillation light output as function of temperature. The single crystal sample was attached to the copper sample holder with the ^241^Am source placed behind the sample inside of a He-flow cryostat. A multi-alkali photomultiplier model 9124A (Electron Tube Enterprises) was used for measurements of scintillation light emitted by the crystal.

The scintillation light yield of CsPbBr_3_ was determined by comparing with the response of reference scintillator LYSO-Ce, known for its fast decay time (33 ns) and high light yield (34000 ph/MeV), both changing insignificantly with cooling^81,82^. Under the assumption of identical light collection efficiency, the measured light output of a crystal ${N}_{x}$ is proportional to the two variables, i.e. absolute light yield ($L{Y}_{x}$) and emission-weighted detector sensitivity ${\varepsilon }_{\lambda }$ ^83–85^:

1 $${N}_{x} \sim L{Y}_{x}\times {\varepsilon }_{\lambda }$$

The emission-weighted detector sensitivity is determined from the spectral sensitivity of the detector $\varepsilon (\lambda )$ and the emission spectra of the crystal $s(\lambda )$:

2 $${\varepsilon }_{\lambda }=\frac{\int \varepsilon (\lambda )s(\lambda )d\lambda }{\int s(\lambda )d\lambda }$$

Thus, the scintillation light yield can be estimated from the measurements of pulse height spectra in the reference LYSO-Ce scintillator and CsPbBr_3_ crystal under study:

3 $$L{Y}_{CsPbBr3}=L{Y}_{LYSO}\times \frac{{N}_{CsPbBr3}}{{N}_{LYSO}}\,\times \frac{{\varepsilon }_{\lambda ,LYSO}}{{\varepsilon }_{\lambda ,CsPbBr3}}.$$

In the second method, we quantified the scintillation response of CsPbBr_3_ using X-ray excitation produced by the Diamond synchrotron in the following way. The synchrotron emits a sequence of X-ray pulses separated by a 2-ns gap. The X-ray pulses excite a sequence of scintillation pulses in the crystals that are then recorded by photodetector. Bearing in mind the mentioned above assumption regarding the invariable light collection efficiency we infer that the detector response integrated over a certain time, and corrected for the emission weighted spectral sensitivity, is proportional to the scintillation light yield of a material. Subsequently, the response of scintillators *N*_*x*_ was integrated over a 20 ns time interval and the light yield of CsPbBr_3_ under X-ray excitation was derived using the formula (3) taking into account the reported non-proportionality of 55% at 12 keV for LYSO-Ce^73^. The theoretical limit for the absolute light yield of the CsPbBr_3_ scintillator is evaluated using semi-empirical model^71,72^. In this case the energy transfer efficiency and luminescence quantum efficiency is assumed to be equal to 1, bringing about the following equation for the absolute light yield of a scintillator:

4 $$LY=\frac{{10}^{6}}{2.35{E}_{g}}{\left[1+0.158\times {10}^{4}\left\{\frac{1}{{\varepsilon }_{\infty }}-\frac{1}{{\varepsilon }_{0}}\right\}\frac{{(h{\nu }_{LO})}^{\frac{3}{2}}}{1.5{E}_{g}}\right]}^{-1}\,({\rm{ph}}/{\rm{MeV}})$$

Here ${E}_{g}\,$ = 2.25 eV is band gap energy, ${\varepsilon }_{0}\,$ = 37^86^ and ${\varepsilon }_{\infty }$ = 1.9^87^ are static and high-frequency relative permittivity of the material, $h{\nu }_{LO}$ = 19 meV^4^ is the maximum energy of LO phonons. Substituting the numerical values into Eq. (4) an upper limit for the light yield of CsPbBr_3_ is found to be equal to 117,000 ph/MeV.
